# Integrating primary care and public health to enhance response to a pandemic

**DOI:** 10.1017/S1463423621000311

**Published:** 2021-06-10

**Authors:** Karen Kinder, Andrew Bazemore, Melina Taylor, Cristina Mannie, Stefan Strydom, Joe George, Felicity Goodyear-Smith

**Affiliations:** 1 Technische Universität Berlin, Berlin, Germany; 2 American Board of Family Medicine, Lexington, Kentucky, USA; 3 Center for Professionalism & Value in Health Care, Washington, DC, USA; 4 Mast Analytics, Claremont, Western Cape, South Africa; 5 Department of General Practice & Primary Health Care, University of Auckland, Auckland, New Zealand

**Keywords:** COVID-19, primary health care, public health

## Abstract

Primary health care (PHC) includes both primary care (PC) and essential public health (PH) functions. While much is written about the need to coordinate these two aspects, successful integration remains elusive in many countries. Furthermore, the current global pandemic has highlighted many gaps in a well-integrated PHC approach. Four key actions have been recognized as important for effective integration.

A survey of PC stakeholders (clinicians, researchers, and policy-makers) from 111 countries revealed many of the challenges encountered when facing the pandemic without a coordinated effort between PC and PH functions. Participants’ responses to open-ended questions underscored how each of the key actions could have been strengthened in their country and are potential factors to why a strong PC system may not have contributed to reduced mortality.

By integrating PC and PH greater capacity to respond to emergencies may be possible if the synergies gained by harmonizing the two are realized.

## Introduction

In October 2018, on the 40th anniversary of the Alma Ata Declaration, representatives from around the globe gathered in Astana, Kazakhstan to declare a renewed focus on primary health care (PHC) acknowledged in the Declaration of Astana (World Health Organization and United Nations Children’s Fund, 2018a). However, a mere 2 years later, the global COVID-19 pandemic exposed persistent and glaring gaps in global aspirations for well-integrated PHC (UHC2030 International Health Partnership, 2020).

One of the commitments proclaimed in the Declaration of Astana is to ‘*build sustainable primary health care*’ which is further elaborated as enhancing ‘*capacity and infrastructure for primary care (PC) – the first contact with health services – prioritizing essential public health functions*’. The Declaration goes further stating, ‘*We will benefit from sustainable PHC that enhances health systems’ resilience to prevent, detect and respond to infectious diseases and outbreaks*’ (World Health Organization and United Nations Children’s Fund, 2018a). Hence, PHC includes both PC and essential public health (PH) functions (see Table 1) (World Health Organization, 2019). While much is written about the need to coordinate these two aspects, successful integration remains elusive in many countries (World Health Organization, 2018b; 2018c; 2019; Rechel, 2020).

**Table 1.** Definitions of primary health care, primary care, and essential public health functions according to the World Health Organization (World Health Organization, 2018b)

**Primary health care.** A whole-of-society approach to health that aims to maximize the level and distribution of health and well-being through three components: (a) primary care and essential public health functions as the core of integrated health services; (b) multisectoral policy and action; and (c) empowered people and communities.
**Primary care.** A key process in the health system that supports first-contact, accessible, continued, comprehensive, and coordinated patient-focused care.
**Essential public health functions**. The spectrum of competences and actions that are required to reach the central objective of public health – improving the health of populations. This document focuses on the core or vertical functions: health protection, health promotion, disease prevention, surveillance and response, and emergency preparedness.

The World Health Organization Resolution on the PHC draft Operational Framework, approved by the World Health Organization Executive Board in January 2020 (World Health Organization, 2019), provides guidance on operationalizing the Astana commitments through strategic and operational levers. It encourages pursuit of ‘*Models of care that promote high-quality, people-centred primary care and essential public health functions as the core of integrated health services throughout the course of life*’ which seem vital to an effective pandemic response.

However, the intersection of PC and PH remains ill-defined and varies by setting (Rechel, 2020). In their Technical Series on PHC, the World Health Organization (WHO) outlines the benefits of coordinating PC and PH and identifies actions which could contribute to successful integration (World Health Organization, 2018c). The pandemic has provided an unexpected lens through which to gauge global readiness to take four key actions recognized as important for effective integration:

1) ‘*Enabling primary care to deliver more protective, promotive, and preventive services to a defined population;*
2) *Improving communication and coordination between public health authorities and PC providers and managers;*
3) *Sharing knowledge and data to evaluate the impact of both individual- and population-focused services on health; and*
4) *Strengthening the surveillance function of primary care and more effectively linking this to public health surveillance*’ (World Health Organization, 2018c).

This paper stems from a multi-national study on the perspectives of PC experts on their country’s response to the pandemic and aims to assess the degree to which PC and PH were integrated in national responses to the current coronavirus pandemic from the study’s free-text responses and learn from successes as well as failures.

## Methods

In an effort to better understand global PHC response to the pandemic, we conducted a survey from 15 April 2020 to 4 May 2020 using a convenience sample pulled from the World Organization of Family Doctors member network, WHO, and other global PHC organization contact lists. Respondents were encouraged to share the survey, so it is not clear how many total recipients received it. The 34-item survey assessed attributes of a strong PC system (see Table 2), national preparedness, and national response strategies to counter the COVID-19 pandemic. Further elaboration on survey methods, validation, and qualitative and quantitative analyses are presented in previously published papers (Goodyear-Smith, *et al.*, 2020, 2021).

**Table 2.** Attributes of primary care assessed in the PHC_COVID survey

Q6. As of 1 January 2020, in your opinion, was affordable, accessible patient-centered primary care available in your country?
Q7. As of 1 January 2020, did the majority of primary care facilities in your country provide comprehensive primary care for acute and chronic conditions, infectious and non-communicable diseases?
Q8. As of 1 January 2020, is there registration/enrollment/assignment of the population to a responsible primary care clinician (e.g. empanelment)? In other words, are most family doctors aware of the population they are responsible for and is the population aware of their responsible primary care clinician whom they would see first to ensure their health care is coordinated?
Q9. As of 1 January 2020, is a referral from a primary care clinician required to seek care from a specialist or hospital (gatekeeper role) except in cases of emergency?
Q15. Is there a unique identifier attached to every person in your health system used to track all health care associated with that person across settings for your whole country (e.g. a Unique Patient Identification Number or UPIN)?
Q16. To what degree do patient-level records (a single record that addresses the multiple conditions a patient presents with) exist in your country?
Q17. Were e-consultations (i.e. including telephone calls, emails, visitations via Skype, etc.) between primary care doctors and patients routinely used prior to the current pandemic?

## Findings

A total of 1035 PC respondents (clinicians, researchers, policy-makers, and other global actors) from 111 countries completed the survey with good representation across all regions and economic levels (see Table 3). Participants’ text responses to open-ended questions in the survey were identified which underscored how each of the four key actions could have been strengthened in their country and are potential factors to why a strong PC system may not have guaranteed reduced mortality (Goodyear-Smith, *et al*., 2020) (see Table 4).

**Table 3.** Summary table of count of respondents by country, World Bank economic tiers and World Health Organization region. Survey responses were captured between 15 April 2020 and 4 May 2020

Country	English	Spanish	Total responses	% Total responses	Economic tier	WHO region
Australia	163		163	15.7%	HIE	WPRO
New Zealand	99		99	9.6%	HIE	WPRO
Mexico	13	65	78	7.5%	UMIE	PAHO
Malaysia	77		77	7.4%	UMIE	PAHO
United States of America	55	1	56	5.4%	HIE	PAHO
Italy	30		30	2.9%	HIE	EURO
Spain	27	2	29	2.8%	HIE	EURO
Trinidad and Tobago	27		27	2.6%	HIE	PAHO
Finland	27		27	2.6%	HIE	EURO
United Kingdom	27		27	2.6%	HIE	EURO
Canada	24		24	2.3%	HIE	PAHO
China	23		23	2.2%	UMIE	WPRO
Switzerland	20		20	1.9%	HIE	EURO
South Africa	16		16	1.5%	UMIE	AFRO
Belgium	15		15	1.4%	HIE	EURO
Estonia	14		14	1.4%	HIE	EURO
United Arab Emirates	14		14	1.4%	HIE	EMRO
Argentina	6	5	11	1.1%	UMIE	PAHO
Brazil	9	1	10	1.0%	UMIE	PAHO
Netherlands	10		10	1.0%	HIE	EURO
Korea, Republic of	10		10	1.0%	HIE	WPRO
India	9		9	0.9%	LMIE	SEARO
Nigeria	9		9	0.9%	LMIE	AFRO
Croatia	9		9	0.9%	HIE	EURO
Singapore	8		8	0.8%	HIE	WPRO
Fiji	8		8	0.8%	HIE	WPRO
Iceland	7		7	0.7%	HIE	EURO
Nepal	7		7	0.7%	LMIE	SEARO
Philippines	7		7	0.7%	LMIE	WPRO
Ghana	7		7	0.7%	LMIE	AFRO
Israel	7		7	0.7%	HIE	EURO
Japan	6		6	0.6%	HIE	WPRO
Myanmar	6		6	0.6%	LMIE	SEARO
Norway	6		6	0.6%	HIE	EURO
Kazakhstan	6		6	0.6%	UMIE	EURO
Chile	4	1	5	0.5%	HIE	PAHO
Thailand	5		5	0.5%	UMIE	SEARO
Turkey	5		5	0.5%	UMIE	EURO
Austria	4		4	0.4%	HIE	EURO
Pakistan	4		4	0.4%	LMIE	EMRO
Sweden	4		4	0.4%	HIE	EURO
Romania	4		4	0.4%	HIE	EURO
Indonesia	4		4	0.4%	UMIE	SEARO
Guyana	4		4	0.4%	UMIE	PAHO
Kenya	4		4	0.4%	LMIE	AFRO
Tanzania, United Republic of	3		3	0.3%	LMIE	AFRO
France	3		3	0.3%	HIE	EURO
Bangladesh	3		3	0.3%	LMIE	SEARO
Colombia	3		3	0.3%	UMIE	PAHO
Taiwan	3		3	0.3%	HIE	WPRO
Greece	3		3	0.3%	HIE	EURO
Germany	3		3	0.3%	HIE	EURO
Jamaica	3		3	0.3%	UMIE	PAHO
Bosnia and Herzegovina	3		3	0.3%	UMIE	EURO
Egypt	3		3	0.3%	LMIE	EMRO
Barbados	2		2	0.2%	HIE	PAHO
Sri Lanka	2		2	0.2%	LMIE	SEARO
Uganda	2		2	0.2%	LIE	AFRO
Kyrgyzstan	2		2	0.2%	LMIE	EURO
Mozambique	2		2	0.2%	LIE	AFRO
Panama	2		2	0.2%	HIE	PAHO
Timor-Leste	2		2	0.2%	LMIE	SEARO
Lebanon	2		2	0.2%	UMIE	EMRO
Costa Rica	2		2	0.2%	UMIE	PAHO
Portugal	2		2	0.2%	HIE	EURO
Georgia	2		2	0.2%	UMIE	EURO
Lesotho	2		2	0.2%	LMIE	AFRO
Jordan	2		2	0.2%	UMIE	EMRO
Saint Kitts and Nevis	2		2	0.2%	HIE	PAHO
Czech Republic	2		2	0.2%	HIE	EURO
Saudi Arabia	2		2	0.2%	HIE	EMRO
Denmark	2		2	0.2%	HIE	EURO
Malawi	2		2	0.2%	LIE	AFRO
Afghanistan	2		2	0.2%	LIE	EMRO
Slovakia	2		2	0.2%	HIE	EURO
Venezuela		2	2	0.2%	UMIE	PAHO
Bhutan	2		2	0.2%	LMIE	SEARO
Zimbabwe	2		2	0.2%	LMIE	AFRO
Cuba	1	1	2	0.2%	UMIE	PAHO
Iraq	1		1	0.1%	UMIE	EMRO
Djibouti	1		1	0.1%	LMIE	EMRO
Botswana	1		1	0.1%	UMIE	AFRO
Macedonia, the former Yugoslav Republic of	1		1	0.1%	UMIE	EURO
Uzbekistan	1		1	0.1%	LMIE	EURO
Rwanda	1		1	0.1%	LIE	AFRO
Honduras		1	1	0.1%	LMIE	PAHO
Ireland	1		1	0.1%	HIE	EURO
Cyprus	1		1	0.1%	HIE	EURO
Saint Vincent and the Grenadines	1		1	0.1%	UMIE	PAHO
Iran, Islamic Republic of	1		1	0.1%	UMIE	EMRO
Ecuador		1	1	0.1%	UMIE	PAHO
Poland	1		1	0.1%	HIE	EURO
Malta	1		1	0.1%	HIE	EURO
Democratic Republic of the Congo	1		1	0.1%	LIE	AFRO
Bolivia	1		1	0.1%	LMIE	PAHO
Hungary	1		1	0.1%	HIE	EURO
Somalia	1		1	0.1%	LIE	EMRO
Bahamas	1		1	0.1%	HIE	PAHO
Antigua and Barbuda	1		1	0.1%	HIE	PAHO
Ethiopia	1		1	0.1%	LIE	AFRO
Algeria	1		1	0.1%	LMIE	AFRO
Latvia	1		1	0.1%	HIE	EURO
Montenegro	1		1	0.1%	UMIE	EURO
Uruguay		1	1	0.1%	HIE	PAHO
Sudan	1		1	0.1%	LIE	EMRO
Vanuatu	1		1	0.1%	LMIE	WPRO
Swaziland/Eswatini	1		1	0.1%	LMIE	AFRO
Viet Nam	1		1	0.1%	LMIE	WPRO
Morocco	1		1	0.1%	LMIE	EMRO
Guinea	1		1	0.1%	LIE	AFRO
Mongolia	1		1	0.1%	LMIE	WPRO
*Total responses*	954	81	1035	100.0%		
						
Legend:		N				
						
*Economic tier*						
Low-income economy	LIE	10				
Low-middle income economy	LMIE	27				
Upper-middle income economy	UMIE	27				
High-income economy	HIE	47				
						
*Region*						
African region	AFRO	17				
Eastern Mediterranean region	EMRO	13				
European region	EURO	36				
Region of the Americas	PAHO	24				
South-East Asian region	SEARO	9				
Western Pacific region	WPRO	12				

*Sources:* https://datahelpdesk.worldbank.org/knowledgebase/articles/906519-world-bank-country-and-lending-groups

https://www.who.int/choice/demography/mortality_strata/en/

**Table 4.** Summary table of survey findings aligned with WHO suggested actions to integrate primary care and public health for pandemic response. Survey responses were captured between 15 April 2020 and 4 May 2020

WHO suggested action	Summary findings
*Action 1*: Enable primary care (PC) service delivery to a defined population	- PC was not considered for pandemic response in several countries - COVID-19 testing, treatment, and management was outsourced to stand-alone drive-through ‘facilities’ or delegated to hospitals - PC clinicians are essential to ensure a country-wide coordinated response
*Action 2*: Improve communication and coordination between public health (PH) and PC	- Clear, consistent, and reliable messaging is needed from national authorities - Updated information as the pandemic changes helps public health and primary care coordinate patient care more effectively
*Action 3*: Improve sharing of individual and population data and knowledge	- Primary care clinicians should be notified of their patients who test positive for treatment or isolation measures if they are not administering testing - Existing treatments and screenings for patients should be maintained and coordinated to adhere to pandemic response plans
*Action 4*: Strengthen the surveillance function of primary care	- Surveillance within the community, including primary care clinicians and community health workers should be utilized, as well as nationally to accurately track and response to outbreaks of viruses/disease - It is essential that a primary care perspective be incorporated into any pandemic plan.

### Action 1: enable PC service delivery to a defined population

While good PC services may have been available, in some countries, these were not utilized or integrated. A respondent from Mexico wrote ‘*A national response to COVID-19 has been focused on hospital services. PHC has not been taken into consideration’*, and from Uzbekistan: ‘*Our response is completely hospital/ER-centric. No one has talked about getting more testing and evaluation by the primary care sector’,* The role of PC in triaging patients and only referring those in need of hospitalization can mitigate the overburdening of emergency and secondary care services.

Not all countries were perceived to have an existing and implemented pandemic plan. From Malaysia, one respondent mentioned ‘*National guidelines for handling the COVID-19 needed to be formulated earlier’*. Countries that had experienced previous pandemics were more prepared: ‘*polyclinic doctors were well prepared and so were the 900 public health private GP clinics because Singapore had experience with SARs’*.

Additionally, the degree to which PC was incorporated into the pandemic plan is unclear. We noted only a weak positive correlation (*R* = 0.1308) between respondents who thought a pandemic plan was utilized and believed their country had a strong PC system (Goodyear-Smith, *et al*., 2020). The need to include PC clinicians in the crafting of such a plan was highlighted as critical to ensure a system-wide coordinated response. In addition, the disconnect between national- and state-level plans was evident in some federal health systems.

### Action 2: improve communication and coordination between PH and PC

Absence of clear and consistent communication between national and local PH entities and the PC community was a theme clearly emergent in survey responses. Two Australian general practitioners noted *‘my information was received late in the overall evolution of the pandemic. Most … came through the media rather than reliable medical and infectious disease centres’* and ‘*information was often passed on by word of mouth’*. From a positive perspective, a respondent from Estonia conveyed ‘*We organised a so-called committee of coronacrisis and the committee provided counselling and needed help for all family practices*’ and in New Zealand ‘*strong leadership jointly by the Prime Minister and by the Director-General of Health on medical & public health matters … [a] clear communication strategy’*.

Several respondents identified poor communication from health authorities to health care delivery sites, such as a comment from an Indian respondent ‘*Confusion in communicating whom we must see and whom we must not’,* and a respondent from Germany stated, ‘*The local administration with a crisis-committee is not aware of coordinating primary care services with hospitals’.* Where there was insufficient governmental guidance ‘*armchair experts filled the void of uncertainty’* which further added to the noise in Australia. A critical role of family doctors is to discern and dispel the plethora of false information, often promulgated through the internet, whether it be regarding the virus or under usual circumstances. As relayed from a respondent from Mexico ‘*there is disinformation of the population spread x social networks’.* One respondent from Trinidad and Tobago identified the importance of consistent communication, ‘*Daily updates are given to the nation by the Ministries of Health, National Security, and Social Welfare’.*

In a void of reliable information, clinicians were left to craft and convey clear messages to patients on the virus and self-care, a function built on the trust and relational continuity central to PC. This is particularly critical where a government response is missing or misguided. As conveyed by one Australian respondent, ‘*We tried to do the right thing initially, moving chairs away from us, telling EVERYONE who came to see us how dangerous the virus was’.* Another respondent noted ‘*On the whole, the public’s response has been excellent but better and more timely information would have provided more trust between the public and politicians’.*

### Action 3: improve sharing of individual and population data and knowledge

The sharing of patient data and records between PC and PH is a long-standing touchpoint. When testing is conducted outside of PC practices, for example, at drive-through test centers, the test results must be communicated to PC clinicians. This enables the family doctor to follow up with the patient on treatment and isolation, as well as proactively assess the family’s situation and contact trace. It also ensures that non-COVID-19 PC services for patients and their families are maintained and coordinated (World Health Organization, 2019).

In many countries, the PH functions of testing, isolation, and tracing are not performed in PC facilities. In Hong Kong, ‘*primary care is essentially private. Hospital care is government (public) and private. So initially no kits available for testing in private’.* Many believed the lack of test kits exacerbated the problem with patients resorting to seeking care directly from hospitals. A Malaysian respondent identified PC testing as key, ‘*if PC clinics do not screen, identify and isolate, COVID transmission will be out of control and increase exponentially’.*

In some settings, integration of PC and PH may require additional training of PC staff in PH interventions, thereby expanding the comprehensiveness of the services PC clinicians are competent to deliver (World Health Organization; 2018b). Such a reorientation of PC to include a population health management approach, with a person-centered focus rather than an orientation toward diseases, can improve the health of an entire community (World Health Organization, 2018a; Rechel, 2020).

### Action 4: strengthen the surveillance function of PC

In some countries, the role of PC in surveillance was emphasized. In India, it was noted that ‘*the existing polio surveillance network and the chain of community health workers has definitely helped in better contact tracin*g’, and in Spain ‘*primary care has played a very important role. All patients (except emergency) are attended initially in the health center, which acts as a filter, and applies a common protocol, and depending on the patient’s clinical situation, the patient remained at home with surveillance and follow-up by primary care professionals’.* In Thailand, ‘*government health facilities recruited professionals from private sectors to help with the increased need for surveillance/support at quarantine sites’.*

However, in other countries, it was believed that the surveillance function of PC should be strengthened. From Afghanistan: ‘*primary health care had the potential to have been used aggressively in community-level surveillance, contact tracing, health promotion and health protection as primary health care facilities are based closer to the community’.*

## Discussion

This pandemic is not over and there are lessons to extract from recent experiences in hopes of altering the trajectory of the next wave (UHC2030 International Health Partnership, 2020). Findings from this study highlight the inadequacy of existing PC and PH integration efforts, the need for including PC stakeholders in the planning process, the role of the PC clinician in communicating a clear message, as well as the necessity for PC clinicians to be involved in surveillance, triaging, and follow-up (see Table 4).

Despite the expressed frustration, there were reports of positive experiences of coordination of PC and PH. Ultimately, integration requires the leadership of PC clinicians and officials supporting PHC to actively inject themselves in the PH solutions which are being turned to.

Acknowledging that health systems vary and there is no ‘one-size-fits-all’ model for how best to integrate PC with essential PH functions, the WHO recommended actions which can facilitate inclusion of PC in the planning for pandemics, the response to mitigate the spread, the treatment and finally, the vaccination efforts to deflect future waves of the virus (World Health Organization, 2018c). The recognition of PC as ‘the first point of contact’ was bypassed by many countries during the pandemic as many initial responses were delivered by hospitals. By integrating PC and PH, greater capacity to respond to emergencies may be possible if the synergies gained by harmonizing the two are realized.
